# Prediction score of indications for whole body computed tomography in blunt trauma patients

**DOI:** 10.1186/cc12232

**Published:** 2013-03-19

**Authors:** H Oda, R Sasaki, A Hagiwara, A Kimura

**Affiliations:** 1 National Center For Global Health and Medicine, Tokyo, Japan

## Introduction

Whole body computed tomography (WBCT) appears to be useful for the early detection of clinically occult injury, although its indications have been controversial. The purpose of this study was to develop a clinical prediction score to clarify the indications for blunt trauma patients with multiple injuries (MI) who require WBCT.

## Methods

We conducted a retrospective study of 173 patients with blunt trauma who underwent WBCT at our emergency center between June 2011 and July 2012. We chose the presence or absence of MI (Injury Severity Score ≥15) in need of surgical intervention as the outcome variable. We used bivariate analyses to identify variables potentially predicting the presentation of MI. The predictor variables were confirmed by multivariate logistic regression analyses. We assigned a score based on the corresponding coefficients.

## Results

**Figure 1:**
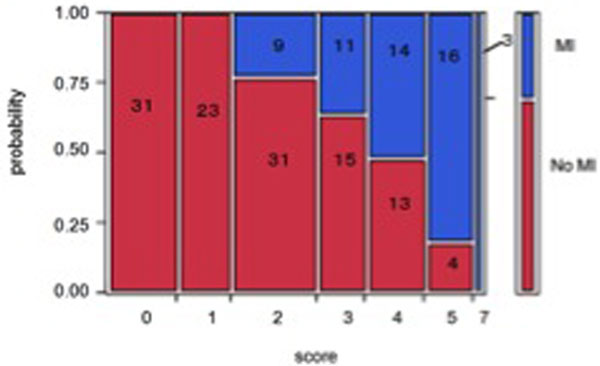
**Prediction versus MI probability (validation group)**.

**Figure 2:**
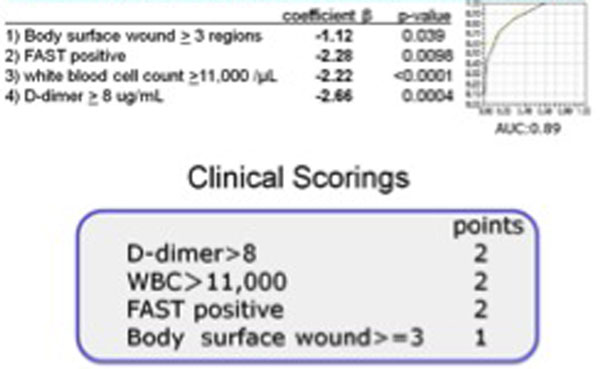
**Clinical scorings**. Multivariate, logistic regression analyses.

Among the 173 patients enrolled, 53 were in the MI group. Four predictors were found to be independently significant by the logistic analysis: (1) body surface wound ≥3 regions, (2) positive focused assessment with sonography for trauma, (3) white blood cell count ≥11,000/µl, and (4) D-dimer ≥8 µg/ml. Score 1 was assigned to predictor (1), score 2 was assigned to predictors (2), (3) and (4). A prediction score was calculated for each patient by adding these scores. The area under the receiver operating characteristic curve was 0.89. No patients with a score of 1 or less had MI (Figures 1 and 2).

## Conclusion

In patients with a score of 1 or 0, the presence of MI is less likely. These patients may not require WBCT, and selective CT scans of body parts based on clinical presentation should be considered.
